# Accurate Free Energy Calculation via Multiscale Simulations Driven by Hybrid Machine Learning and Molecular Mechanics Potentials

**DOI:** 10.1021/acs.jctc.5c00598

**Published:** 2025-07-04

**Authors:** Xujian Wang, Xiongwu Wu, Bernard R. Brooks, Junmei Wang

**Affiliations:** † Department of Pharmaceutical Sciences and Computational Chemical Genomics Screening Center, School of Pharmacy, 6614 University of Pittsburgh, Pittsburgh, Pennsylvania 15261, United States; ‡ Laboratory of Computation Biology, National Heart, Lung and Blood Institute, 2511 National Institutes of Health, Bethesda, Maryland 20892, United States

## Abstract

This work develops a hybrid machine learning/molecular mechanics (ML/MM) interface integrated into the AMBER molecular simulation package. The resulting platform is highly versatile, accommodating several advanced machine learning interatomic potential (MLIP) models while providing stable simulation capabilities and supporting high-performance computations. Building upon this robust foundation, we developed new computational protocols to enable pathway-based and end point-based free energy calculation methods utilizing ML/MM hybrid potential. In particular, we proposed an ML/MM-compatible thermodynamic integration (TI) framework that adequately addressed the challenge of applying MLIPs in TI calculations due to its indivisible nature of energy and force. Our results demonstrated that the hydration free energies calculated using this framework achieved an accuracy of 1.0 kcal/mol, outperforming the traditional approaches. Moreover, ML/MM enables more precise sampling of conformational ensembles for improved end point-based free energy calculations. Overall, our efficient, stable, and highly compatible interface not only broadens the application of MLIPs in multiscale simulations but also enhances the accuracy of free energy calculations from multiple aspects. By introducing a novel ML/MM-compatible thermodynamic integration framework, we offered a novel foundation for combining advanced multiscale simulation methodologies with highly accurate free energy calculation techniques, thereby opening new avenues and providing a robust theoretical framework for future developments in this field.

## Graphical abstract

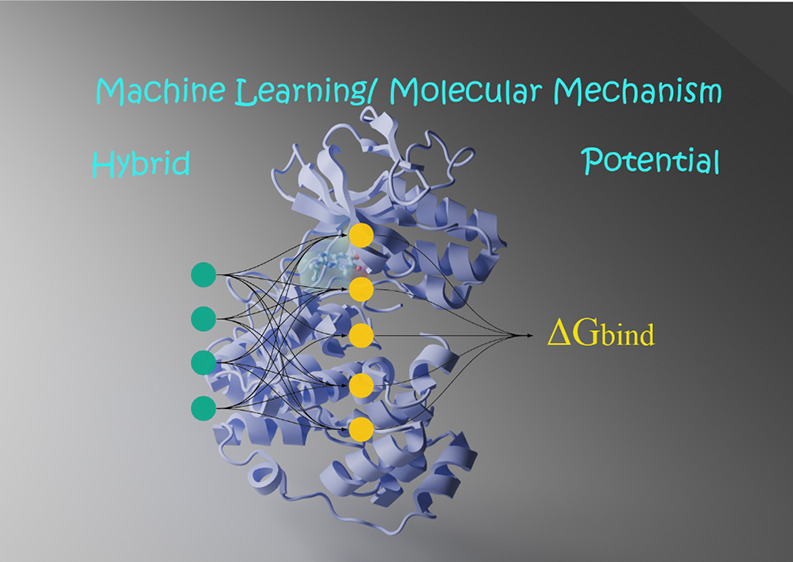

## 1 Introduction

In the field of molecular dynamics (MD) simulations, much work has contributed to improving molecular mechanics force fields (MMFF) to achieve a higher accuracy in reproducing experimental metrics. Efforts include extending general small molecule force fields,^1,2^ developing new protein force fields,^3–6^ and creating force fields for other biomolecules, such as DNA^7,8^ and lipids.^9,10^ However, it remains a challenge to accurately reproduce quantum mechanism (QM) results with classic MMFF, especially when chemical reactions are involved. To overcome this challenge, a feasible solution is to combine the computationally efficient MMFF method with the accurate QM methods. In the 1970s, Warshel Arieh and Levitt Michael^11^ proposed quantum mechanics/molecular mechanics (QM/MM) molecular dynamics, which applies a QM model to describe the essential part of the system (such as atoms involved in a chemical reaction), and MMFF to describe the rest of the system.^12,13^ This hybrid simulation technology allows for investigation of electronic structures and chemical reactions in large systems.

In recent years, many efforts have been made to improve QM/MM MD simulations. For example, the electrostatic embedding approach was proposed to accurately calculate the electrostatic interaction,^14,15^ and polarizable force fields have been introduced to account for interatomic polarization effects.^16–18^ Furthermore, improvements in long-range interaction processing have enhanced the accuracy of simulations.^19,20^ However, the computational cost has been the primary limitation to the broad application of these hybrid simulation techniques. The bottleneck in QM/MM studies is QM calculation, which remains very time-consuming.

However, Behler and Parrinello^21^ and Csányi and co-workers^22,23^ proposed machine learning interatomic potentials (MLIPs) over a decade ago as an alternative to traditional quantum mechanical approaches. MLIPs accelerate calculations by being trained on machine learning algorithms to reproduce ab initio quantities such as potential energies and atomic forces, thereby avoiding time-consuming quantum mechanical calculations. Building on this framework, many modern MLIPs have emerged by incorporating various advanced artificial intelligence (AI) techniques.^24–33^ Take ANI-2x^27^ as an example, which was trained on data from ωB97X/6-31G­(d)^34,35^ calculations and achieved near density functional theory^36,37^ (DFT) accuracy while maintaining computational efficiency comparable to molecular mechanics. Given their accuracy and performance, MLIPs could potentially serve the role of ab initio models in the simulation of biomolecular systems with QM/MM. Incorporation of MLIPs into a molecular dynamics engine to develop a brand-new multiscale simulation technique is appealing given their near-QM level of accuracy and near-MM level of efficiency. Thus, machine learning/molecular mechanics molecular dynamics (ML/MM MD) represents a promising alternative for biomolecular simulations.

A great effort has been put into implementing ML/MM in molecular simulations of biomolecular systems,^38–42^ laying a strong foundation for future advancements. To achieve an accurate and rigorous description of the energetics of a system divided by the ML and MM regions, researchers have introduced several refinements, including long-range interaction corrections^43–45^ and electrostatic embedding schemes.^41,46–50^ Building upon these developments, it is timely to extend the application of the ML/MM approach to more demanding tasks such as accurate free energy calculations. This research direction is attractive considering the dual advantages of MLIP models, high computational efficiency and near ab initio-level accuracy, which makes them particularly suitable for long-time scale simulations to produce diverse, statistically meaningful conformational ensembles. Recent studies have demonstrated the feasibility of using ML/MM for free energy calculations.^51,52^ However, the current computational protocols of free energy calculations using either free energy perturbation (FEP) or thermodynamic integration (TI), cannot be applied to the current ML/MM hybrid potentials. Thus, the systematic development of a new theory of pathway-based free energy calculation for MM/ML is necessary.

In this work, we extended the AMBER simulation platform^53,54^ by introducing a versatile ML/MM interface, designed to be compatible with a wide range of MLIP models. We demonstrated the robustness of the implementation through benchmark tests and performed comprehensive performance profiling to evaluate its computational efficiency. Next, we developed a systematic theoretical framework for thermodynamic integration calculations using the extended ML/MM platform. To validate this new framework, we conducted hydration-free energy calculations for a set of structurally diverse molecules. We further evaluated the advantages of ML/MM in the end point binding free energies of numerous protein–ligand complexes, as we expected that ML/MM could produce more meaningful conformations. By leveraging advances in efficient and accurate conformational sampling together with a rigorous and compatible theoretical framework for pathway-based free energy calculations, we expect our work to pave the way for more reliable free energy predictions by using hybrid ML/MM potentials. Moreover, we envision that it will serve as a foundational step toward the development of more advanced alchemical free energy calculation methodologies.

## 2 Theory

### 2.1 Theoretical Details for the ML/MM Approach

The ML/MM approach shares strong conceptual similarities with the well-established QM/MM framework.^11,55^ The theoretical foundation of ML/MM based on mechanical embedding has reached a mature stage,^38–40^ in which the total energy of the system is partitioned into three components: the ML region, the MM region, and their mutual interaction:

$$E_{\mathrm{total}}=E_{\mathrm{ML}}+E_{\mathrm{MM}}+E_{\mathrm{ML}‐\mathrm{MM}}$$ 1

Here, **E**_ML_ is obtained using MLIPs, while **E**_MM_ is computed via classical molecular mechanics force field (MMFF) equations.^56^

For the ML–MM interaction term, in order to ensure compatibility with a broad range of MLIP models, we adopt the widely used mechanical embedding scheme,^38,39^ which is both efficient and broadly supported. This scheme describes the nonbonded interactions between the ML and MM regions using a combination of Coulombic and Lennard–Jones (LJ) potentials:

$$E_{\mathrm{ML}‐\mathrm{MM}}=\sum_{i\in \mathrm{MM}}\sum_{j\in \mathrm{ML}}\frac{q_{i}q_{j}}{\left|R_{i}^{\mathrm{MM}}-R_{j}^{\mathrm{ML}}\right|}+\sum_{i\in \mathrm{MM}}\sum_{j\in \mathrm{ML}}[(\frac{A}{(|R_{i}^{\mathrm{MM}}-R_{j}^{\mathrm{ML}}|{)}^{12}})-(\frac{B}{(|R_{i}^{\mathrm{MM}}-R_{j}^{\mathrm{ML}}|{)}^{6}})]$$ 2

In this equation, **R**_*i*_^MM^ and **R**_*j*_^ML^ denote the atomic coordinates of the atoms in the MM and ML regions, respectively. The terms *q*_*i*_ and *q*_*j*_ represent the atomic partial charges. The parameters *A* and *B* represent preparametrized van der Waals parameters.

### 2.2 Theory of Thermodynamic Integration in ML/MM Systems

TI is a robust method for estimating free energy changes and has board applications.^57–62^ Traditionally, TI calculations follow the equation below:^63^

$$\Delta G=G_{\lambda =1}-G_{\lambda =0}=\int_{0}^{1}{\left\langle \frac{\partial V}{\partial \lambda }\right\rangle }_{\lambda }d\lambda$$ 3

The basic principle of TI is to introduce a parameter λ to gradually perturb the system’s potential energy (*V*), facilitating the transformation of the system from the initial state (*G*_λ=0_) to the final state (*G*_λ=1_). In practice, several windows at different λ values are used to numerically estimate the integral:

$$\Delta G=\sum_{i}w_{i}{\left\langle \frac{\partial V}{\partial \lambda }\right\rangle }_{i}$$ 4

Here, *w*_*i*_ represents the weighting factor associated with each window. In this context, potential energy ⟨*V*⟩_*i*_ becomes a central quantity of interest. Because the potential is typically computed using molecular mechanics force fields, it can be further decomposed into bonded and nonbonded components:

$${\left\langle \frac{\partial V_{\mathrm{tot}}}{\partial \lambda }\right\rangle }_{i}={\left\langle \frac{\partial V_{\mathrm{bonded}}}{\partial \lambda }\right\rangle }_{i}+{\left\langle \frac{\partial V_{\mathrm{non}‐\mathrm{bonded}}}{\partial \lambda }\right\rangle }_{i}$$ 5

When computing solvation free energies or absolute binding free energies (ABFEs), covalent bonds are preserved throughout the simulation. Under this treatment, the bonded interactions are identical in both the initial and final states, that is, the bonded term (⟨*V*_bonded_⟩) remains invariant and is unaffected by the perturbation; thus, the change in potential energy arises solely from the nonbonded interactions (⟨*V*_nonbonded_⟩). The same treatment is introduced in the TI scheme for an MM/ML hybrid potential, which describes the region of interest using MLIPs. The key advantage of employing MLIPs in free energy calculations lies in its near ab initio level accuracy.

When bonded terms are omitted, the potential energy in the ML/MM scheme can be rewritten as follows:

$${\left\langle \frac{\partial V_{\mathrm{tot}}}{\partial \lambda }\right\rangle }_{i}={\left\langle \frac{\partial V_{\mathrm{non}‐\mathrm{bonded}}}{\partial \lambda }\right\rangle }_{i}={\left\langle \frac{\partial V_{\mathrm{MM}‐\mathrm{ML},\mathrm{non}‐\mathrm{bonded}}}{\partial \lambda }\right\rangle }_{i}+{\left\langle \frac{\partial V_{\mathrm{ML}‐\mathrm{ML},\mathrm{non}‐\mathrm{bonded}}}{\partial \lambda }\right\rangle }_{i}$$ 6

The corresponding free energy expression can be derived by reformulating the conventional TI equation.^63^ Because the solvation free energy calculation shares conceptual similarities with ABFE calculations, we use the solvation free energy calculation as an illustrative example:

$$\begin{array}{rl} \Delta G_{\mathrm{solvation}} & =\sum_{i}w_{i}\left[{\left\langle \frac{\partial V}{\partial \lambda }\right\rangle }_{\mathrm{wat},i}-{\left\langle \frac{\partial V}{\partial \lambda }\right\rangle }_{\mathrm{gas},i}\right]\\ & =\sum_{i}w_{i}\left[{\left\langle \frac{\partial V_{\mathrm{MM}‐\mathrm{ML},\mathrm{non}‐\mathrm{bonded}}}{\partial \lambda }\right\rangle }_{\mathrm{wat},i}+{\left\langle \frac{\partial V_{\mathrm{ML}‐\mathrm{ML},\mathrm{non}‐\mathrm{bonded}}}{\partial \lambda }\right\rangle }_{\mathrm{wat},i}-{\left\langle \frac{\partial V_{\mathrm{ML}‐\mathrm{ML},\mathrm{non}‐\mathrm{bonded}}}{\partial \lambda }\right\rangle }_{\mathrm{gas},i}\right] \end{array}$$ 7

Notably, current MLIP models are trained to reproduce the total potential energy and atomic forces, without explicitly separating the nonbonded terms within the ML region (i.e., *V*_ML‑ML,non‑bonded_). If one attempts to introduce a λ to directly perturb this term, then the bonded interactions would inevitably be affected as well, potentially introducing significant errors into the free energy calculation.

To address this challenge, our ML/MM TI scheme omits the perturbation of nonbonded interactions within the ML region. Instead, we introduce an additional energy term, termed the reorganization energy (see below), to compensate for this omission. As a result, *V*_MM‑ML,non‑bonded_ becomes the only term subjected to perturbation during the TI process.

Because we do not introduce λ to perturb the ML region, both ${\left\langle \frac{\partial V_{\mathrm{ML}‐\mathrm{ML},\mathrm{non}‐\mathrm{bonded}}}{\partial \lambda }\right\rangle }_{\mathrm{wat}}$ and ${\left\langle \frac{\partial V_{\mathrm{ML}‐\mathrm{ML},\mathrm{non}‐\mathrm{bonded}}}{\partial \lambda }\right\rangle }_{\mathrm{gas}}$ are always equal to zero. However, in eq 7, it is clear that ${\left\langle \frac{\partial V_{\mathrm{ML}‐\mathrm{ML},\mathrm{non}‐\mathrm{bonded}}}{\partial \lambda }\right\rangle }_{\mathrm{wat}}-{\left\langle \frac{\partial V_{\mathrm{ML}‐\mathrm{ML},\mathrm{non}‐\mathrm{bonded}}}{\partial \lambda }\right\rangle }_{\mathrm{gas}}$ describes the energy difference due to conformational changes of the molecule between the water and gas phases. To account for the disappearance of terms ${\left\langle \frac{\partial V_{\mathrm{ML}‐\mathrm{ML},\mathrm{non}‐\mathrm{bonded}}}{\partial \lambda }\right\rangle }_{\mathrm{wat}}$ and ${\left\langle \frac{\partial V_{\mathrm{ML}‐\mathrm{ML},\mathrm{non}‐\mathrm{bonded}}}{\partial \lambda }\right\rangle }_{\mathrm{gas}}$ , we introduce a correction term to offset this effect:

$$\Delta G_{\mathrm{reorg}}=\langle E_{\mathrm{ML}}\rangle_{\mathrm{wat}}-\langle E_{\mathrm{ML}}\rangle_{\mathrm{gas}}$$ 8

We define the energy raised from the conformational change as the reorganization energy (Δ*G*_reorg_), which is calculated as the difference between the average energies of the solvated- and gas-phase conformational ensembles.

Thus, the TI scheme we proposed can solve the challenge for a ML/MM hybrid potential due to the inseparable nature of energy terms in MLIPs, and it is largely compatible with the traditional ML/MM approach. This new TI scheme was then validated by using hydration free energy calculations for a set of structurally diverse compounds.

$$\Delta G_{\mathrm{solvation}}=\sum_{i}w_{i}{\left\langle \frac{\partial V_{\mathrm{MM}‐\mathrm{ML},\mathrm{non}‐\mathrm{bonded}}}{\partial \lambda }\right\rangle }_{\mathrm{wat},i}+\Delta G_{\mathrm{reorg}}$$ 9

## 3 Results and Discussion

### 3.1 Design and Benchmarking of a Flexible MLIP Interface

Mainstream MD simulation packages have begun to embrace the ML/MM approach in recent years,^38–40^ and the rapid growth of ML/MM development highlights its potential as an appealing tool to revolutionize multiscale simulation techniques. AMBER, a widely used MD engine, has already integrated the ANI model and implemented a state-of-the-art electrostatic embedding scheme for ML/MM simulations.^41^ However, current support is limited to the ANI-series models. To further enhance the flexibility and expand the applicability of ML/MM in AMBER, we implemented a general ML/MM interface in the Amber 2023 package, primarily within SANDER, an efficient, CPU-based MD engine. This interface lays the foundation for incorporating a broader range of advanced MLIP models into the AMBER platform.

In order to expand flexibility and compatibility with various MLIP models while ensuring high performance, we have implemented the interface using the LibTorch library, which enables efficient MLIP inference and force calculations. To further support these goals, we adopted an asynchronous workflow (Scheme S1) in which conventional MD calculations are executed on CPUs and MLIP inference runs concurrently on GPUs. Additionally, our implementation utilizes the mechanical embedding scheme—a method commonly employed in QM/MM frameworks,^55^ which allows users to explicitly define the ML region while treating the remaining atoms with classical force fields. Under this framework, we have successfully integrated multiple MLIP models into SANDER, including the ANI series (ANI-1x,^26^ ANI-1ccx,^25^ and ANI-2x^27^) and the MACE series^29^ (MACE-OFF23­(S), MACE-OFF23­(M), and MACE-OFF23­(L)). Overall, this design significantly accelerates simulations by fully leveraging heterogeneous hardware resources, providing a robust and versatile platform for future MLIP developments.

Notably, the major limitation of traditional QM/MM simulations is their high computational cost, which restricts simulation speeds to the picosecond per day range. In contrast, our ML/MM framework delivers a significant speed-up. Specifically, using the ANI-2x model, most simulations reach over 2 ns/day (Figure 1A), and MACE-OFF23­(S) achieves around 1.5 ns/day (Figure 1B). For comparison, traditional QM/MM simulations for the same systems are limited to no more than 6 ps/day (Figure 1C), meaning our ML/MM approach runs roughly 1000–2000 times faster. This efficiency brings near ab initio accuracy while greatly improving the computational performance. All reported simulations used a 1 fs (fs) time step; when bonds involving hydrogen are constrained using the SHAKE algorithm,^64^ the time step can be extended to 2 fs, effectively doubling the simulation performance for systems where detailed hydrogen dynamics are less critical.

**Figure 1:**
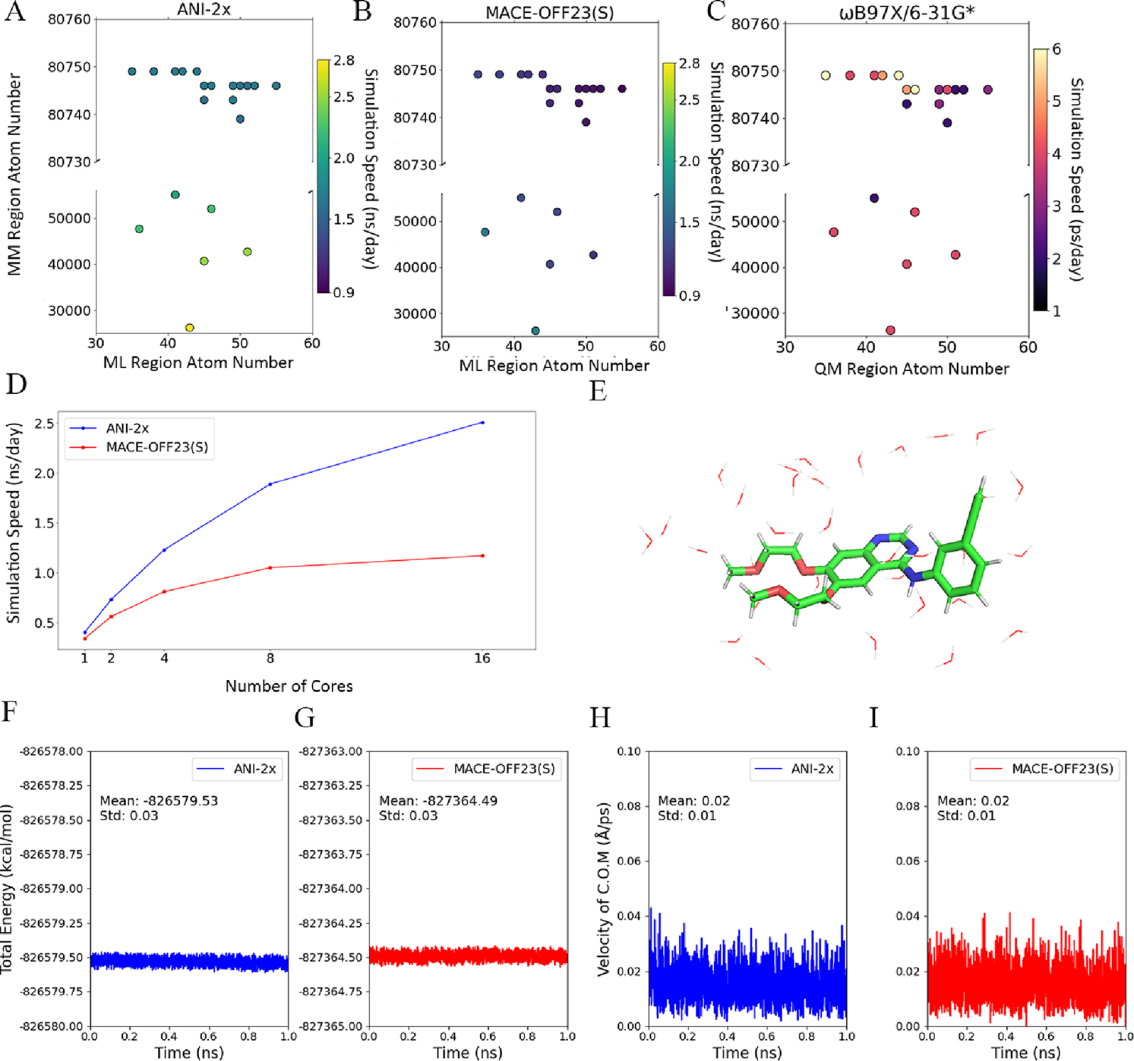
Comprehensive Evaluation of Performance and Robustness in ML/MM Simulations. (A,B) compare the performance of ML/MM implementations using ANI-2x and MACE-OFF23­(S) in different protein–ligand complexes. (C) illustrates the simulation performance of a QM/MM approach with QM calculated at the ωB97X/6-31G* level. (D) demonstrates how the number of CPU cores influences ML/MM simulation speed in a protein–ligand complex system (PDB ID: 2ZFF; 42,685 atoms in the MM region and 51 atoms in the ML region). (E) Energy evolution for a system containing erlotinib in water. (F,G) Time profiles of the total energy during the simulations across the different approaches, while (H,I) time profiles of the center-of-mass velocity.

Although ML/MM already demonstrates significant performance advantages over traditional QM/MM approaches, variability among models and the complexities of efficiently distributing workloads across GPU and CPU resources remain critical challenges. Therefore, we conducted workload tests to evaluate the performance scaling. The results show that increasing CPU cores up to 16 enhances the overall simulation speed (Figure 1D). Notably, the ANI-2x model benefits more from additional cores than MACE-OFF23­(S), suggesting that GPU calculations using ANI-2x are faster, and its bottleneck lies in CPU-based MM computations. In contrast, MACE-OFF23­(S) reaches a performance plateau at 8 cores, suggesting MACE-OFF23­(S) is a GPU-demanding model primarily due to its large parameter set and a versatile architecture.^28–30^ Despite its slower performance, ongoing improvements, such as reducing model parameters^29^ and adopting the JAX MD framework,^65^ may boost MACE’s speed. Overall, ML/MM enables nanosecond-time scale simulations with near ab initio accuracy, representing a substantial enhancement over traditional QM/MM approaches.

Beyond performance, the reliability of the ML/MM approach is critical. To assess the robustness of our ML/MM approach, we simulated erlotinib,^66^ an EGFR inhibitor, in water under microcanonical (NVE, i.e., constant number of particles, volume and total energy of the system) conditions. The system consists of 151 atoms (Figure 1E): 52 atoms from erlotinib define the ML region, while the remaining 99 atoms represent 33 water molecules. This configuration, with the ML region comprising approximately 34.4% of the total atoms, enables a comprehensive evaluation of its energy contribution. We employed ANI-2x and MACE-OFF23­(S) as the MLIP models in our ML/MM MD calculations. The average energies obtained from the ML/MM simulations were −826,579.53 kcal/mol for ANI-2x (Figure 1F) and −827,364.49 kcal/mol for MACE-OFF23­(S) (Figure 1G), each with a standard deviation of 0.03 kcal/mol, a small fluctuation that is very close to the QM/MM value of 0.02 kcal/mol as reported in a previous publication.^20^ The slight discrepancies are likely due to numerical errors. To further assess the simulation stability, we analyzed key momentum parameters. The center-of-mass velocity remained effectively negligible at 0.02 for both ANI-2x and MACE-OFF23­(S) (Figure 1H,I, respectively). In addition, the computed translational and rotational energies support this stability: the translational energy remained below 0.15 kcal/mol (Figure S1), while the rotational energy was even lower, 0.02 kcal/mol for ANI-2x and 0.03 kcal/mol for MACE-OFF23­(S). These observations confirm that the ML/MM approach robustly conserves both the momentum and energy as well as faithfully reproduces the system’s thermodynamic behavior in accordance with the laws of thermodynamics.

### 3.2 Pathway-Based Hydration Free Energy Calculations

Mobley and Guthrie reported experimental hydration free energy data for hundreds of molecules.^67^ Notably, when estimated using the MMFF method with the conventional TI protocol, the hydration free energies of these molecules exhibited deviations of approximately ±1.5 kcal/mol.^58–60^ From this data set, we carefully selected 30 compounds containing six elements of C, H, O, N, F, and Cl to represent a diverse array of functional groups, including ketones, amines, and halides (Figure 2A). We then applied our ML/MM-compatible TI approach (see Section 2.2) to predict the hydration free energies using ANI-2x and MACE-OFF23­(S) in conjunction with GAFF2.^2^

**Figure 2:**
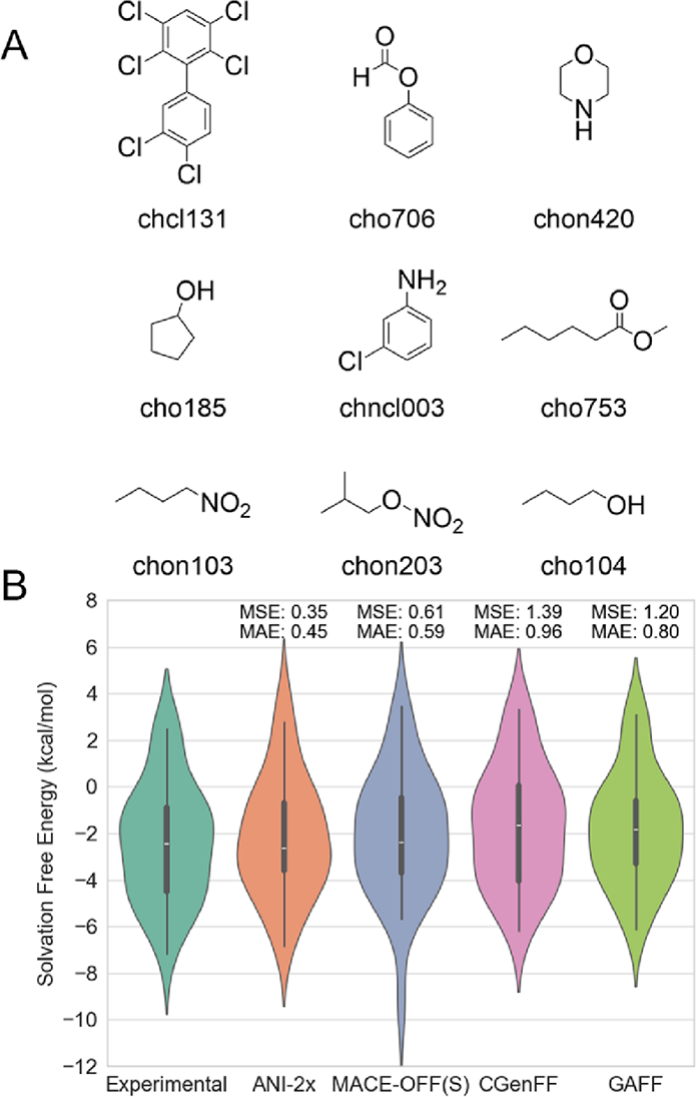
Prediction of hydration free energy using ML/MM approaches and classical force fields. (A) Structures of several compounds used for the TI calculations. (B) Final results obtained from the TI calculations.

Figure 2B illustrates the prediction accuracy of different models. Note that the results for CGenFF^1^ and GAFF^2^ were directly obtained from previous publications.^59,67^ In summary, the overall data distributions of ANI-2x and MACE-OFF23­(S) are relatively similar, with nearly the same mean absolute error (MAE) of 0.45 and 0.59 kcal/mol (Figure 2B), respectively, which are significantly lower than those obtained using either CGenFF (0.96 kcal/mol) or GAFF (0.80 kcal/mol). To our surprise, the MLIP models demonstrated slightly higher accuracy compared to MMFF. However, it is worth noting that in the ML/MM approach, interatomic forces were still described by GAFF2, while intermolecular interactions were calculated at the MLIP level. This discrepancy might lead to consistency issues between the two components. After all, ANI-2x and MACE-OFF23­(S) was trained to reproduce high-accuracy DFT energetics and forces, whereas GAFF2 ^2^ and TIP3P water^68^ were developed to reproduce both quantum mechanics and experimental data. Additionally, the quartile line distribution and mean square error (MSE) indicate that the hydration free energies estimated by ANI-2x and MACE-OFF23­(S) are closer to the experimental data. All of these results indicate that our postulated theory regarding ML/MM demonstrates its comparability to the TI approach in a novel way. Traditional TI, however, employs a gradual scaling-down method to reduce intramolecular interactions, which may also affect interactions between water and the molecule. This creates a highly coupled system; while our approach aims to reasonably decouple these interactions, further efforts are needed to estimate the coupling effects in TI calculations, thereby enhancing the accuracy of ML/MM TI calculations.

### 3.3 Leveraging ML/MM for End Point-Based Free Energy Calculations

Biological macromolecules are essential molecules that carry out complex functions in organisms.^69,70^ Applying ML/MM to the simulation of these macromolecules can enhance our understanding of their mechanisms with near ab initio accuracy at the atomistic level.

We selected six well-studied protein–ligand complexes^71,72^ for our analysis and conducted ML/MM MD simulations on these systems. Over 5 ns simulations, both proteins (Figure 3A,B) and ligands (Figure 3C,D) exhibited only minor fluctuations demonstrating excellent stability of our approach in extended simulations and its high potential applicability to real-world tasks.^73^ To quantitatively assess the quality of the sampled ensembles with our method, we computed B-factors for each structure and compared them to experimental data. In most cases, the computed B-factors correlated well with experimental values, with Pearson correlation coefficients greater than 0.5 (Figure S2). The only exception was the Myeloid cell leukemia 1 protein (PDB ID: 4HW3), which showed a correlation coefficient of 0.18. This lower correlation can be attributed to the fact that the original PDB entry is a multimeric protein, whereas our simulations were performed on only a monomeric unit (Figure S3). The altered environment, which replaces protein–protein interactions with solvent interactions, may account for the discrepancy in dynamics. Furthermore, we generated B-factor maps for the protein (PDB ID: 4GIH) using both experimental and calculated data. The experimental and calculated B-factors were mapped to the protein structure for comparison. As shown in Figures 3E–G and S4, most figures are essentially similar, which underscores the robustness of our ML/MM approach in capturing the essential dynamic behavior of these complexes. Moreover, the excellent conformational sampling provided by ML/MM ensures that the collected structures more accurately reflect the true dynamics of biomacromolecules, thereby facilitating us to correctly capture their complex behaviors.

**Figure 3:**
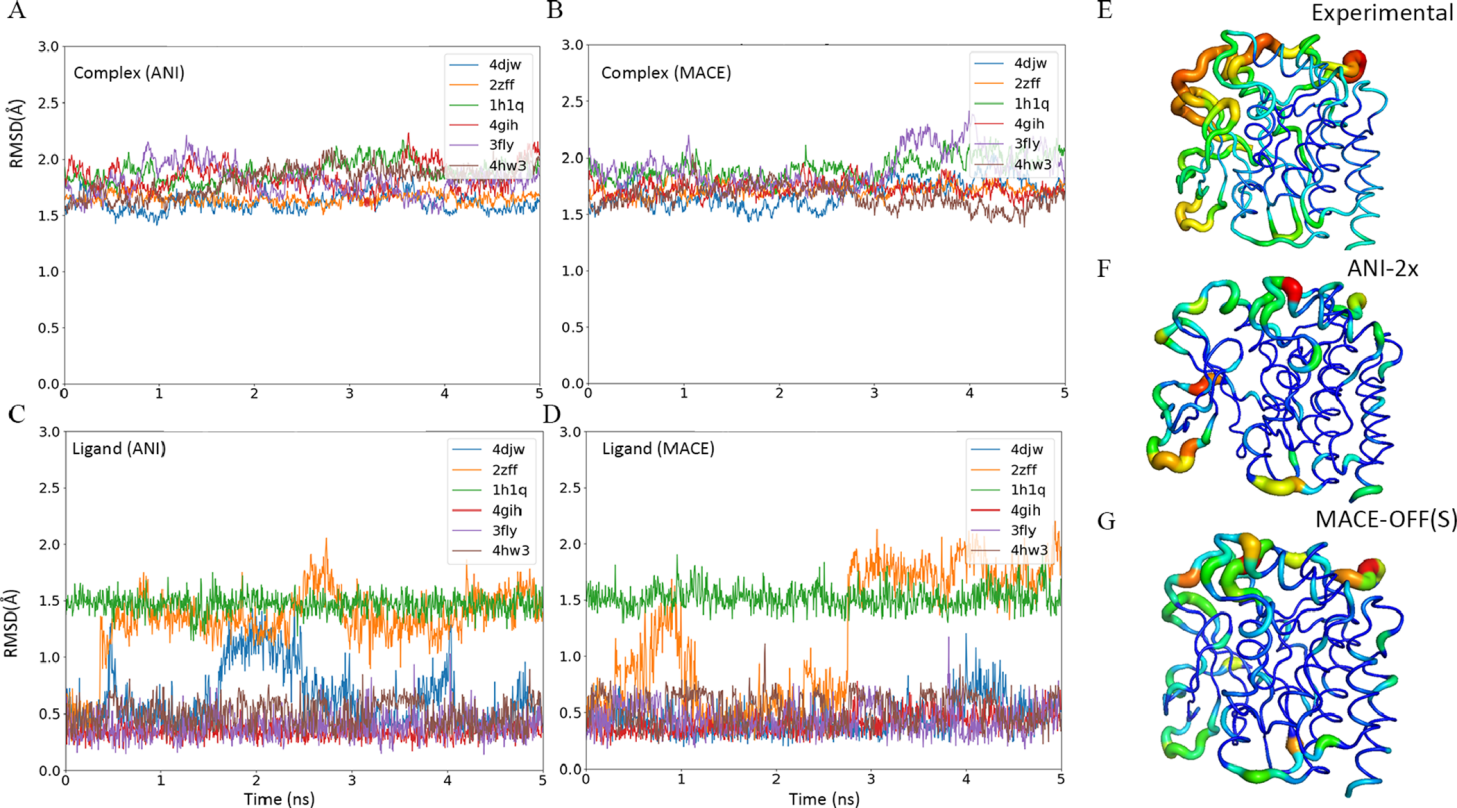
Protein–ligand simulation using an ML/MM approach. (A,B) root-mean-square deviation (RMSD) of the entire protein–ligand complex calculated using ANI-2x and MACE-OFF23­(S), respectively. (C,D) Ligand RMSD based on results from the two MLIPs. (E–G) B-factor color-mapped structures, where the B-factors are derived from the crystal structure and our simulation, respectively.

In addition, this superior conformational sampling capability enables the ML/MM approach to accurately capture thermodynamically meaningful conformations, which in turn enhances the performance of end point-based free energy calculations such as MM-PBSA, a widely used method for predicting protein–ligand binding affinities.^74,75^ To evaluate this protocol, we examined the binding of CDK2 to 19 different ligands.^71,72^ First, ML/MM MD was employed to sample the complex conformations, and then, MM-PBSA was used to analyze the free energies of the obtained structures. Binding free energies derived from the ensembles sampled with MACE achieved an RMSE of 0.65 kcal/mol and an *R* ^2^ of 0.59, outperforming conventional MD which yielded 0.68 kcal/mol and 0.54, respectively. The ANI-2x model produced slightly inferior results with an RMSE of 0.77 kcal/mol and an *R* ^2^ of 0.36. Detailed MD sampling protocols and the results of free energy decomposition into different energetic terms are provided in Tables S1–S3. Unlike pathway-based free energy calculation methods, this end point-based approach can be applied directly to ML/MM trajectories without modifying its fundamental theoretical framework. Consequently, the improved binding free energy calculation accuracy is mainly due to the improved quality of the conformational ensembles sampled by ML/MM. It is expected that the combination of ML/MM sampling with MM-PBSA end point free energy analysis has great applications in elucidating the binding mechanisms of protein and nucleic acid targets.

## 4 Conclusion

In this study, we enhanced the Sander program in the AMBER software suite by developing a versatile and flexible ML/MM interface and systematically benchmarked its robustness. This interface paves the way for integrating more advanced MLIP models in the future, helping to bridge the gap between MLIP development and applications to real-world problems. Importantly, to promote the application of ML/MM in free energy calculations, we proposed a novel TI framework to overcome the challenge of the indivisible nature of energy and force in current MLIP models, ensuring theoretical consistency with the underlying architectures. Building on this advancement, we envision that this framework can also be applied to another widely used free energy calculation method, FEP. This integration of ML/MM and free energy calculation enabled by our framework is promising for more complicated applications, particularly in relative binding free energy (RBFE) calculations. However, one challenge in RBFE calculations that applies multiscale simulation methods is to capture topological changes in molecular structures to enable accurate interaction calculations. Although this challenge remains open, the theoretical consistency and flexibility of our framework provide a solid foundation for advancing ML/MM-based alchemical free energy calculations and broadening their applicability to rigorous binding free energy estimation. In addition to pathway-based free energy methods, widely used end point approaches such as MM-PBSA can also benefit from the ability of ML/MM hybrid potentials to provide long-time scale, high-quality conformational sampling. These advantages pave new avenues for more accurate protein engineering, rational drug design, and a deeper understanding of macromolecular behavior in complex scenarios.

In summary, ML/MM represents a promising direction for the future of molecular simulations. We anticipate that ML/MM can be extended beyond its current capabilities to support more complex applications such as nonadiabatic molecular dynamics and the exploration of chemical reactions. Future work will likely focus on the further description of energetics of ML/MM systems using advanced electrostatic embedding schemes, incorporating long-range interactions into MLIP models, and expanding the overall functionality of ML integration. These developments will enable ML/MM to tackle increasingly complex molecular systems and simulation scenarios.

## Supplementary Material



## Data Availability

The software developed in this work is available at https://github.com/ClickFF/MLMM4AMBER. All benchmarking data, including results from NVE simulations, protein–ligand complex simulations, and free energy calculations, can be accessed athttps://zenodo.org/records/15101823.

## Notes

Computational details; validation of conservation laws using translational and rotational energy; B-factors derived from ML/MM MD simulations; structure of myeloid cell leukemia 1 protein and B-factor color-mapped structures (PDF)
