# Supplementary material for: Accurate Free Energy Calculation via Multiscale Simulations Driven by Hybrid Machine Learning and Molecular Mechanics Potentials
Source: J Chem Theory Comput. 2025 Jul 4;21(14):6979–87. doi: 10.1021/acs.jctc.5c00598 (PMC12288004; doi:10.1021/acs.jctc.5c00598)
Supplement: Supplementary file 1 [file ct5c00598_si_001.pdf]

Supporting Information: Accurate Free Energy Calculation via Multiscale Simulations Driven by Hybrid Machine Learning and Molecular Mechanics Potentials

Xujian Wang,† Xiongwu Wu,*,‡ Bernard R. Brooks,‡ and Junmei Wang*,†

†*Department of Pharmaceutical Sciences and Computational Chemical Genomics Screening Center, School of Pharmacy, University of Pittsburgh, Pittsburgh, Pennsylvania 15261, United States*

‡*Laboratory of Computation Biology, National Heart, Lung and Blood Institute, National Institutes of Health, Bethesda, MD, USA*

E-mail: wuxw@nhlbi.nih.gov; junmei.wang@pitt.edu

Computational Details

ML/MM simulation

In this study, MD simulations were conducted using our internal ML/MM module, based on the AMBER23 source code,[1,2] implemented within the molecular simulation engine SANDER. The simulations configuration were described below. All computation results were obtained using the NVIDIA L40s and the Intel Xeon Platinum 8462Y+.

The protein was modeled using the AMBER14SB force field,[3] and the TIP3P water model[4] was used to model water molecules. Ligands were parameterized using the GAFF2 force field,[5] with partial atomic charges assigned by the ABCG2 method[6] in Antechamber. The systems were constructed using *tleap*, a tool from the AMBERTOOLS23 software suite. The solute molecule was soaked in a TIP3P water box with the smallest distance is at least 10 Ångstrom (Å) between the edges of water box and any solute atom. Na^+ and Cl^- ions were added to neutralize the system and and make the salt concentration about 0.15 M.

All simulations began with an energy minimization step, which employed 1,000 cycles of the steepest descent method, followed by 9,000 cycles of the conjugate gradient method, for a total of 10,000 cycles. Bond lengths involving hydrogen atoms were constrained using the SHAKE algorithm,[7] and a non-bonded cutoff distance of 9.0 Å was applied. The minimization was considered converged when the root-mean-square (RMS) gradient of the potential energy dropped below 0.0001 kcal/mol·Å.

ML/MM simulation for conservation law validation

To establish a closed system and verify the laws of conservation of energy and momentum, we placed only a few water molecules around the ligand, rather than constructing a PBC system. The ligand-water system was minimized in vacuum using a cutoff distance of 999 Å. After minimization, the ligand was designated as the ML region, while the surrounding water molecules were treated using the MMFF. The integration timestep was set to 0.1 femtoseconds, and the simulation ran for 10,000,000 steps, corresponding to a total simulation time of 1 nanosecond. No restraints or constraints were applied to the system. A cutoff distance of 999 Å was

maintained, and the simulation was carried out in the NVE ensemble. To prevent errors from automatic removal of center-of-mass motion in SANDER, the removal frequency (nscm) was set to 999,999,999.

Thermodynamic integration calculation

The system building and minimization steps were described earlier. The TI simulation protocol closely followed that of the protein-ligand complex simulations, beginning with cMD pre-equilibration, followed by the ML/MM TI simulations. For TI simulations, the ligand atoms were defined as both the TI atoms and the ML region atoms. We used 9 windows for both the decharging and de-van der Waals stages, resulting in a total of 18 λ windows. The λ and $klambda$ values were set according to the AMBER 2023 Reference Manual. All other parameters used in the MD simulations were the same as those for the protein-ligand complex simulations.

For the reorganization energy calculation, the cutoff distance in the gas phase was set to 999 Å. In the water phase, the system was pre-equilibrated under cMD conditions, followed by ML/MM simulations. Both phases used a timestep of 1 fs and ran for 1,000,000 steps. Trajectories for the ML/MM simulations were saved every 1,000 steps, resulting in 1,000 frames, which were then used by MLIPs to calculate the average energy difference.

Protein-ligand complex simulation using ML/MM approach

The system building and minimization are described as above. After minimization, the system were simulate using cMD condition without any atom described using MLIPs to reach equilibrium, the integration timestep was set to 1 femtosecond (fs), and the simulations were run for a total of 1,000,000 steps, corresponding to 1 nanosecond of simulation time. The temperature was regulated at 298 K using the Langevin thermostat with with a friction coefficient (gamma_ln) of 5.0 ps^{-1}. Pressure was maintained at 1.01325 bar using the Berendsen barostat with a taup of 2.0 ps. Periodic boundary conditions were enforced to mimic an infinite system, and a nonbonded cutoff distance of 9.0 Å was utilized to calculate van der Waals and electrostatic interactions. Bond lengths involving hydrogen atoms were constrained using the SHAKE

algorithm.

The ML/MM simulation was restarted from it, using nearly the same parameters. While, the ligands were set as ML region and treated using MLIPs, the bond within ML region were no longer constrained by SHAKE algorithm and the total simulation time is 5,000,000 steps, results in 5 nanosecond of simulation time.

MMPBSA calculation

The system building and minimization are described as above. After minimization, the system were simulate using cMD condition without any atom described using MLIPs to reach equilibrium, the integration timestep was set to 1 fs, and the simulations were run for a total of 5,000,000 steps, corresponding to 5 nanosecond of simulation time. Trajectory snapshots were saved every 1,000 steps, resulting in 5000 frames in total. The temperature was regulated at 298 K using the Langevin thermostat with with a friction coefficient (gamma_ln) of 5.0 ps^{-1}. Pressure was maintained at 1.01325 bar using the Berendsen barostat with a taup of 2.0 ps. Periodic boundary conditions were enforced to mimic an infinite system, and a nonbonded cutoff distance of 9.0 Å was utilized to calculate van der Waals and electrostatic interactions. Bond lengths involving hydrogen atoms were constrained using the SHAKE algorithm.

The MM-PBSA key parameters of this protocol includes: 500 snapshots evenly collected from 5 ns NTP simulations for MM-PBSA analysis using the one-trajectory protocol;[8] the polar solvation energies were calculated with the interior and exterior dielectric constants of 1 and 80, respectively; the conformational entropic term, -TS, was estimated using the WSAS approach.[9] More details on MM-PBSA calculations are described somewhere else.[10]

QM/MM calculation

The system setup and minimization processes are described above using the AMBER format. ParmEd tools in AmberTools were used to convert these files into a GROMACS-readable format for subsequent performance tests. The software used in this work includes GROMACS 2024.02[11] and CP2K 2024.02,[12] where GROMACS handles the molecular mechanics (MM) calculations, and CP2K is responsible for the density functional theory (DFT) calculations.

All settings for the MM region in these simulations are identical to those described above. For the quantum mechanics (QM) settings, we used the mechanical embedding method, with van der Waals parameters and partial charges derived from the classical force field. The QM calculations were performed at the ωB97X/6-31G* level of theory, with all other parameters set to the default values in CP2K 2024.02.

ML/MM interface implementation

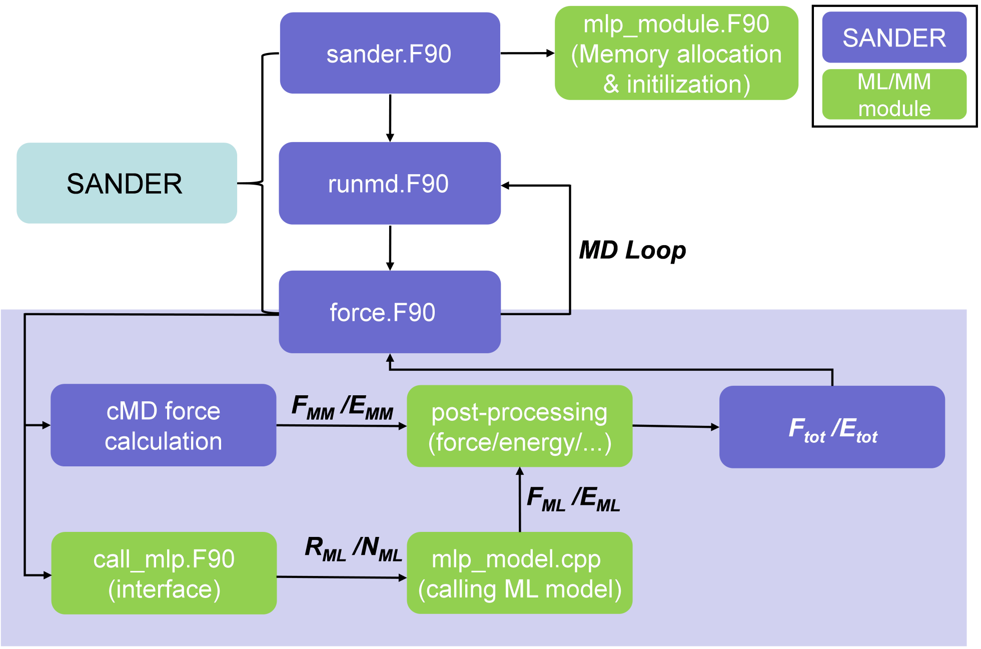

Scheme 1: The implementation details of ML/MM module in SANDER MD engine in Amber. This module is initialized alongside SANDER. Once the MD loop begins, the ML model is invoked through an intrinsic Fortran/C++ interface, facilitating direct communication between the ML algorithms and the core MD routines. The outputs generated by the ML model are then meticulously processed, then they are incorporated into the SANDER mainstream.

Validation of conservation laws

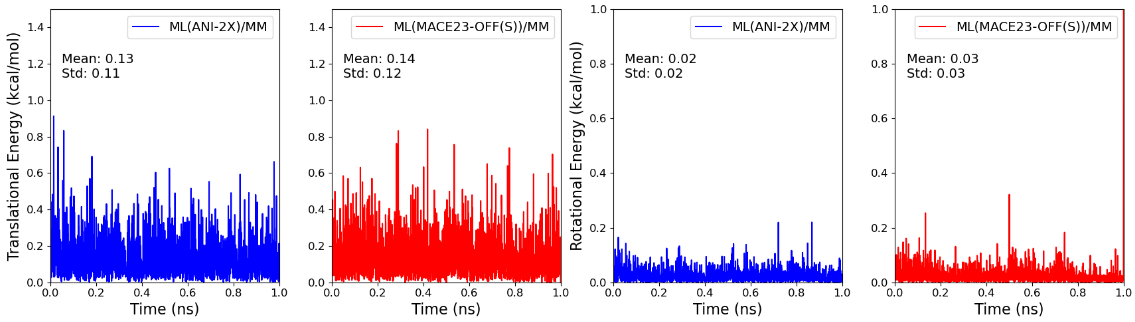

Figure S1: The translational and rotational energies in both conventional MD simulations and ML/MM MD simulations.

Protein ligand complex simulation

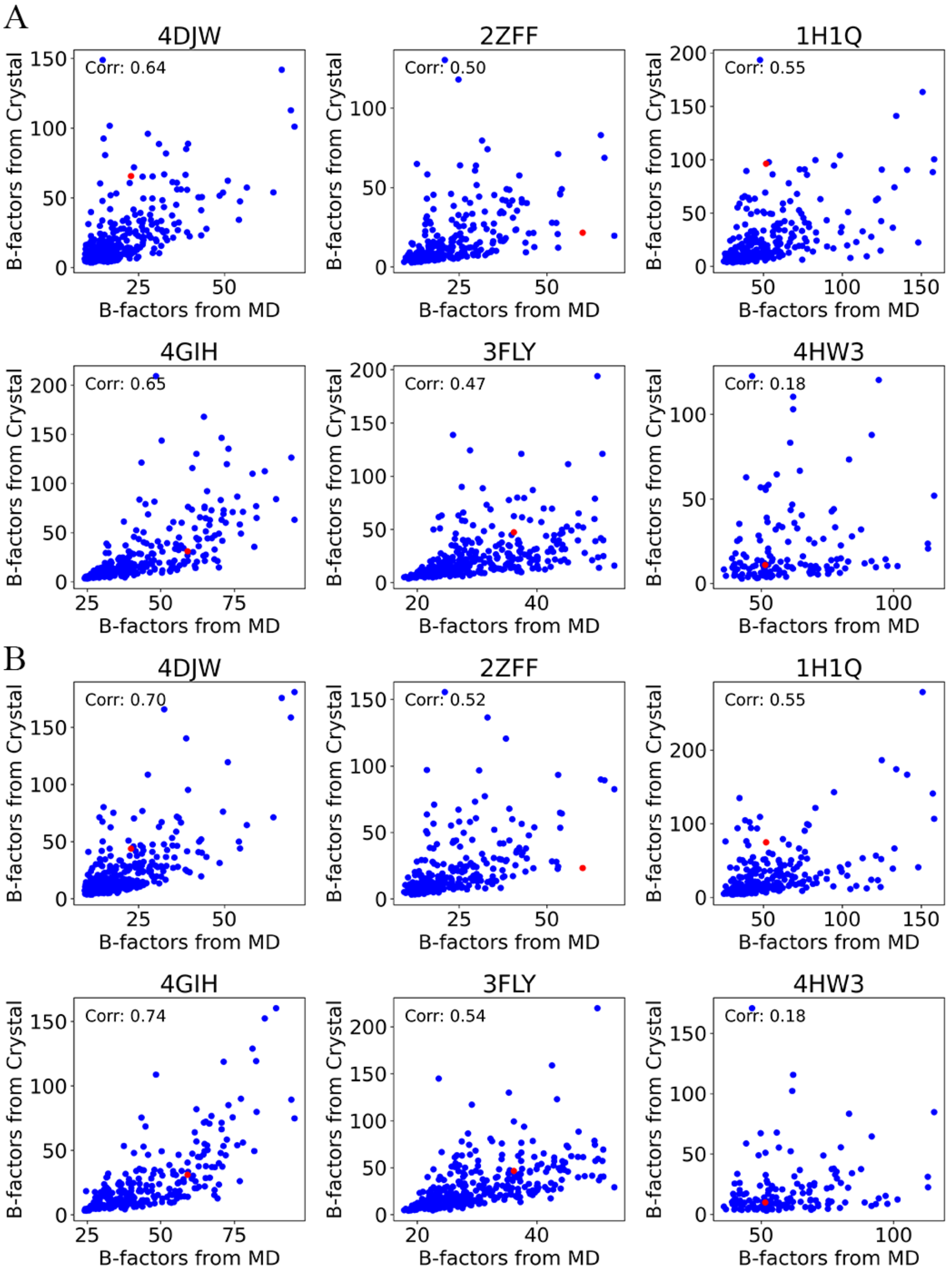

Figure S2: The B-factors derived from ML/MM MD simulations. (A) and (B) display the B-factors obtained from ML/MM MD simulations using the ANI-2x and MACE-OFF23-small models, respectively.

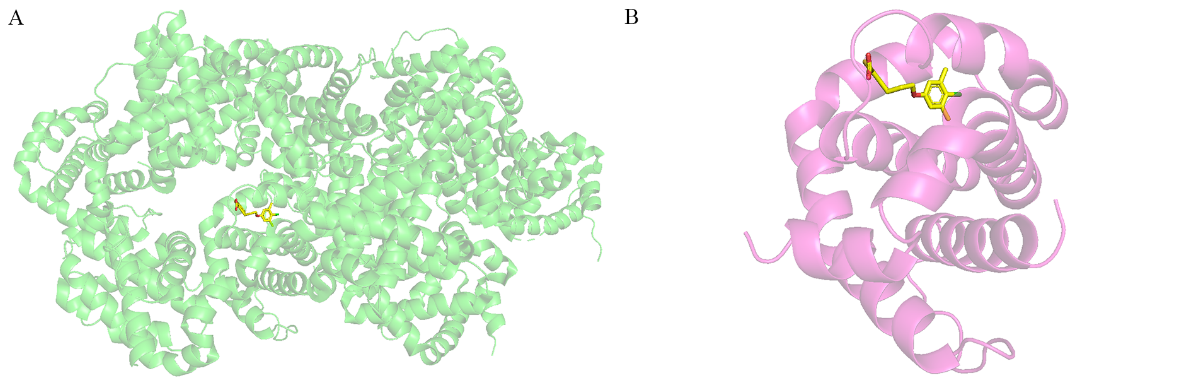

Figure S3: Structure of myeloid cell leukemia 1 protein (PDB ID: 4HW3) as a multimer (A) and as a biological monomer (B).

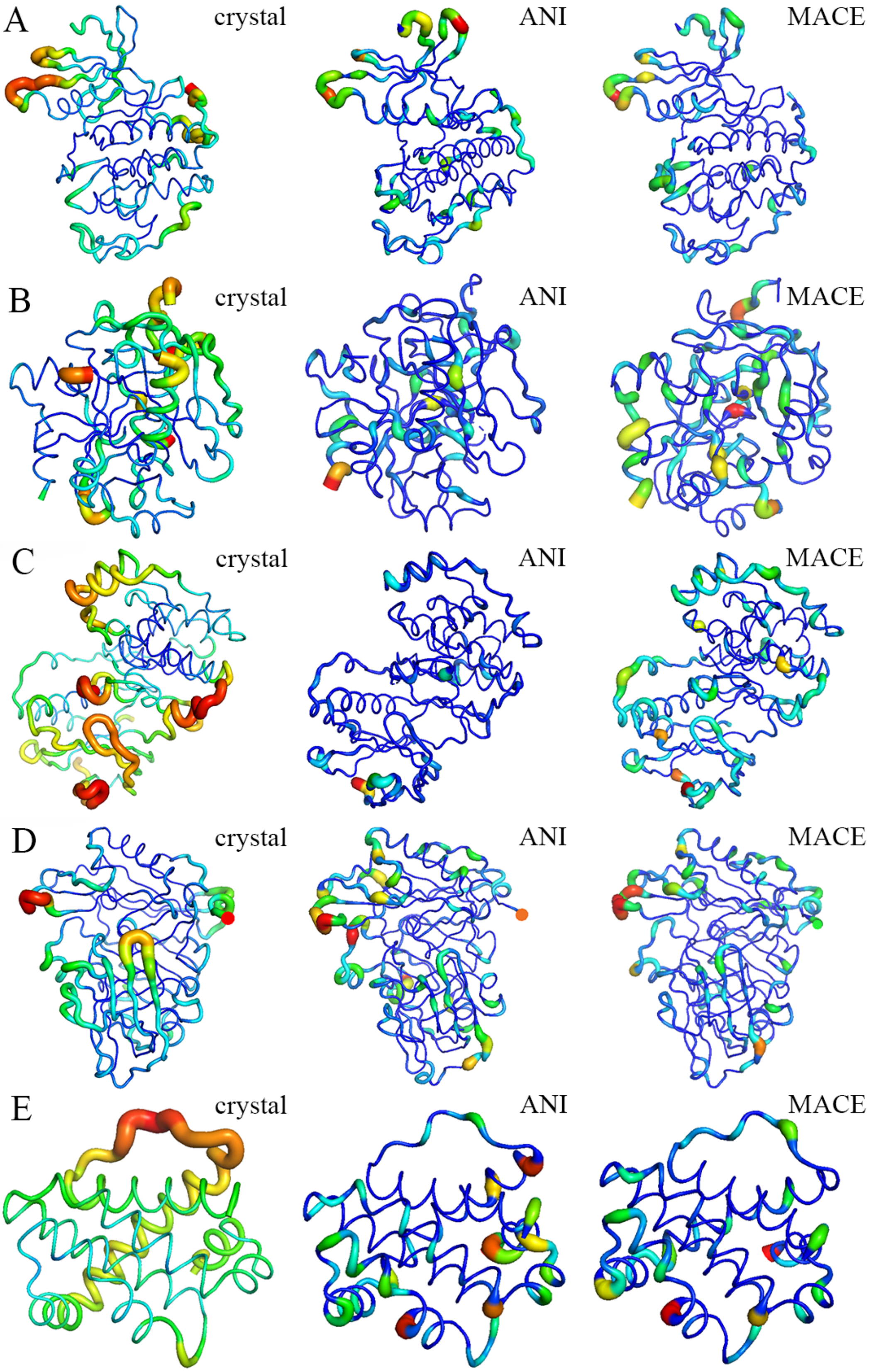

Figure S4: B-factor color-mapped structures. PDB IDs for (A) to (E) are: 1H1Q, 2ZFF, 3FLY, 4DJW, 4HW3.

Table S1: MM-PBSA binding free energy decomposition based on snapshots generated from cMD sampling. Here, ΔE_{VDW} and ΔE_{EEL} represent the van der Waals and electrostatic molecular mechanics terms, respectively. $\Delta G_{\text{sol, PB}}$ and $\Delta G_{\text{sol, SA}}$ correspond to the polar and nonpolar contributions to the solvation free energy, respectively. The TS term denotes the conformational entropy, with calculations performed at a temperature of 25 °C.

**Comp Name**	$\Delta G_{\text{binding}}$ (Kcal/mol)	ΔE_{VDW} (Kcal/mol)	ΔE_{EEL} (Kcal/mol)	$\Delta G_{\text{sol, PB}}$ (Kcal/mol)	$\Delta G_{\text{sol, SA}}$ (Kcal/mol)	TS (Kcal/mol)	$\Delta G_{\text{MM-PBSA}}$ (Kcal/mol)
1	-6.51	-36.99 ± 0.10	-24.19 ± 0.06	33.49 ± 0.15	-2.64 ± 0.00	-20.19 ± 0.03	-10.15 ± 0.12
2	-8.20	-49.69 ± 0.30	-24.71 ± 0.15	38.45 ± 0.13	-3.56 ± 0.00	-22.32 ± 0.01	-17.19 ± 0.22
7	-7.23	-39.95 ± 0.19	-24.67 ± 0.45	33.89 ± 0.11	-2.98 ± 0.00	-20.71 ± 0.03	-13.00 ± 0.48
8	-7.57	-42.37 ± 0.02	-24.55 ± 0.04	34.43 ± 0.02	-3.22 ± 0.00	-21.01 ± 0.03	-14.69 ± 0.06
9	-8.08	-44.94 ± 0.22	-22.82 ± 0.14	34.20 ± 0.08	-3.33 ± 0.00	-21.81 ± 0.01	-15.08 ± 0.30
10	-7.57	-42.98 ± 0.15	-27.61 ± 0.44	40.77 ± 0.07	-3.29 ± 0.01	-21.82 ± 0.04	-11.30 ± 0.31
15	-7.69	-50.13 ± 0.12	-23.89 ± 0.12	38.93 ± 0.12	-3.77 ± 0.01	-22.40 ± 0.05	-16.46 ± 0.17
18	-6.71	-52.28 ± 0.22	-23.49 ± 0.13	39.91 ± 0.08	-3.90 ± 0.00	-23.45 ± 0.03	-16.32 ± 0.14
20	-8.73	-51.56 ± 0.12	-30.30 ± 0.28	45.45 ± 0.17	-3.84 ± 0.00	-23.13 ± 0.02	-17.12 ± 0.21
21	-7.84	-51.60 ± 0.16	-23.35 ± 0.04	39.98 ± 0.07	-3.89 ± 0.00	-22.66 ± 0.03	-16.20 ± 0.20
22	-7.87	-52.57 ± 0.04	-24.70 ± 0.10	39.93 ± 0.13	-3.96 ± 0.01	-23.22 ± 0.03	-18.08 ± 0.14
25	-9.77	-47.66 ± 0.15	-42.96 ± 0.17	44.35 ± 0.23	-3.63 ± 0.00	-22.44 ± 0.04	-27.46 ± 0.04
26	-8.44	-51.27 ± 0.07	-26.64 ± 0.09	39.60 ± 0.25	-3.84 ± 0.01	-22.64 ± 0.02	-19.52 ± 0.24
30	-9.82	-51.01 ± 0.09	-30.73 ± 0.28	46.57 ± 0.31	-4.04 ± 0.01	-23.14 ± 0.04	-16.07 ± 0.12
31	-9.55	-54.34 ± 0.13	-28.94 ± 0.06	45.62 ± 0.06	-4.05 ± 0.01	-24.16 ± 0.01	-17.55 ± 0.10
33	-9.11	-51.79 ± 0.23	-36.86 ± 0.13	50.94 ± 0.19	-4.08 ± 0.01	-23.02 ± 0.03	-18.77 ± 0.09
34	-9.81	-51.36 ± 0.16	-33.42 ± 0.33	43.98 ± 0.03	-3.80 ± 0.00	-23.09 ± 0.03	-21.51 ± 0.47
36	-9.14	-53.84 ± 0.23	-30.81 ± 0.14	43.37 ± 0.08	-4.05 ± 0.01	-23.52 ± 0.01	-21.81 ± 0.23
37	-9.14	-54.48 ± 0.13	-25.07 ± 0.33	39.60 ± 0.12	-4.20 ± 0.01	-23.93 ± 0.03	-20.22 ± 0.26
38	-8.90	-53.09 ± 0.11	-27.64 ± 0.27	41.93 ± 0.08	-3.92 ± 0.00	-23.13 ± 0.03	-19.59 ± 0.38

Table S2: MM-PBSA binding free energy decomposition based on snapshots generated from ANI-2x/MM MD sampling.

**Comp Name**	$\Delta G_{\text{binding}}$ (Kcal/mol)	ΔE_{VDW} (Kcal/mol)	ΔE_{EEL} (Kcal/mol)	$\Delta G_{\text{sol, PB}}$ (Kcal/mol)	$\Delta G_{\text{sol, SA}}$ (Kcal/mol)	TS (Kcal/mol)	$\Delta G_{\text{MM-PBSA}}$ (Kcal/mol)
1	-6.51	-33.95 ± 0.15	-27.10 ± 0.26	34.14 ± 0.19	-2.71 ± 0.00	-19.24 ± 0.04	-10.37 ± 0.15
2	-8.20	-41.38 ± 0.21	-19.34 ± 0.26	31.72 ± 0.26	-3.36 ± 0.00	-20.61 ± 0.02	-11.73 ± 0.08
7	-7.23	-40.34 ± 0.05	-21.66 ± 0.16	31.10 ± 0.36	-2.84 ± 0.00	-20.86 ± 0.01	-12.88 ± 0.21
8	-7.57	-43.18 ± 0.03	-20.61 ± 0.16	31.00 ± 0.13	-3.06 ± 0.00	-21.58 ± 0.02	-14.27 ± 0.25
9	-8.08	-45.84 ± 0.07	-20.67 ± 0.01	30.20 ± 0.06	-3.26 ± 0.00	-22.26 ± 0.03	-17.31 ± 0.12
10	-7.57	-43.34 ± 0.14	-27.72 ± 0.16	37.63 ± 0.22	-3.21 ± 0.00	-21.67 ± 0.05	-14.97 ± 0.27
15	-7.69	-49.55 ± 0.22	-26.30 ± 0.23	42.82 ± 0.41	-3.67 ± 0.01	-22.50 ± 0.03	-14.20 ± 0.35
18	-6.71	-51.19 ± 0.17	-23.79 ± 0.05	39.97 ± 0.13	-3.87 ± 0.01	-23.00 ± 0.03	-15.89 ± 0.08
20	-8.73	-48.55 ± 0.27	-36.16 ± 0.24	48.16 ± 0.11	-3.77 ± 0.00	-22.24 ± 0.01	-18.08 ± 0.09
21	-7.84	-51.06 ± 0.26	-23.21 ± 0.12	42.79 ± 0.38	-3.80 ± 0.00	-22.71 ± 0.00	-12.57 ± 0.29
22	-7.87	-55.59 ± 0.21	-25.75 ± 0.14	46.67 ± 0.07	-3.85 ± 0.00	-24.31 ± 0.02	-14.21 ± 0.14
25	-9.77	-45.67 ± 0.21	-49.38 ± 0.19	51.54 ± 0.16	-3.57 ± 0.00	-22.42 ± 0.02	-24.65 ± 0.13
26	-8.44	-50.96 ± 0.12	-28.74 ± 0.06	42.32 ± 0.25	-3.75 ± 0.00	-22.91 ± 0.02	-18.22 ± 0.27
30	-9.82	-51.06 ± 0.17	-27.25 ± 0.22	43.40 ± 0.12	-3.98 ± 0.00	-22.90 ± 0.02	-15.98 ± 0.15
31	-9.55	-51.92 ± 0.11	-32.80 ± 0.23	48.68 ± 0.33	-3.87 ± 0.00	-22.89 ± 0.03	-17.02 ± 0.20
33	-9.11	-50.45 ± 0.04	-29.81 ± 0.19	47.55 ± 0.09	-4.03 ± 0.00	-23.02 ± 0.03	-13.72 ± 0.24
34	-9.81	-48.84 ± 0.07	-38.52 ± 0.29	53.60 ± 0.10	-3.64 ± 0.00	-22.47 ± 0.02	-14.93 ± 0.31
36	-9.14	-52.49 ± 0.08	-39.98 ± 0.38	53.63 ± 0.46	-3.94 ± 0.01	-23.21 ± 0.03	-19.57 ± 0.28
37	-9.14	-51.27 ± 0.15	-31.01 ± 0.24	44.75 ± 0.09	-3.92 ± 0.00	-23.27 ± 0.06	-18.17 ± 0.24
38	-8.90	-52.64 ± 0.14	-32.42 ± 0.19	49.25 ± 0.21	-3.89 ± 0.01	-22.70 ± 0.02	-16.99 ± 0.17

Table S3: MM-PBSA binding free energy decomposition based on snapshots generated from MACE-OFF(S)/MM MD sampling.

**Comp Name**	$\Delta G_{\text{binding}}$ (Kcal/mol)	ΔE_{VDW} (Kcal/mol)	ΔE_{EEL} (Kcal/mol)	$\Delta G_{\text{sol, PB}}$ (Kcal/mol)	$\Delta G_{\text{sol, SA}}$ (Kcal/mol)	TS (Kcal/mol)	$\Delta G_{\text{MM-PBSA}}$ (Kcal/mol)
1	-6.51	-35.87 ± 0.09	-27.85 ± 0.05	38.75 ± 0.19	-2.69 ± 0.00	-19.46 ± 0.04	-8.19 ± 0.23
2	-8.20	-46.58 ± 0.15	-19.51 ± 0.15	33.28 ± 0.10	-3.39 ± 0.01	-21.44 ± 0.02	-14.76 ± 0.12
7	-7.23	-38.77 ± 0.07	-23.30 ± 0.08	34.14 ± 0.21	-2.87 ± 0.01	-20.15 ± 0.04	-10.64 ± 0.22
8	-7.57	-43.99 ± 0.18	-19.45 ± 0.21	29.98 ± 0.12	-3.08 ± 0.00	-21.67 ± 0.02	-14.87 ± 0.16
9	-8.08	-45.93 ± 0.23	-21.81 ± 0.15	32.53 ± 0.11	-3.23 ± 0.00	-22.18 ± 0.02	-16.25 ± 0.28
10	-7.57	-43.82 ± 0.32	-28.92 ± 0.21	38.04 ± 0.15	-3.16 ± 0.00	-21.78 ± 0.04	-16.08 ± 0.21
15	-7.69	-49.95 ± 0.03	-23.69 ± 0.15	38.80 ± 0.07	-3.66 ± 0.01	-22.51 ± 0.02	-15.99 ± 0.21
18	-6.71	-51.15 ± 0.42	-23.19 ± 0.15	39.09 ± 0.23	-3.82 ± 0.00	-23.00 ± 0.06	-16.08 ± 0.35
20	-8.73	-48.76 ± 0.06	-34.75 ± 0.16	48.34 ± 0.13	-3.80 ± 0.00	-22.17 ± 0.00	-16.80 ± 0.04
21	-7.84	-50.33 ± 0.08	-22.50 ± 0.29	38.89 ± 0.21	-3.77 ± 0.01	-22.59 ± 0.02	-15.12 ± 0.12
22	-7.87	-54.80 ± 0.10	-25.53 ± 0.20	45.54 ± 0.07	-3.76 ± 0.00	-23.87 ± 0.04	-14.68 ± 0.21
25	-9.77	-45.94 ± 0.13	-40.04 ± 0.13	46.82 ± 0.32	-3.60 ± 0.01	-21.98 ± 0.02	-20.79 ± 0.26
26	-8.44	-50.77 ± 0.17	-27.42 ± 0.28	41.73 ± 0.14	-3.71 ± 0.01	-22.72 ± 0.03	-17.45 ± 0.14
30	-9.82	-54.04 ± 0.15	-34.44 ± 0.35	52.69 ± 0.22	-3.89 ± 0.00	-23.83 ± 0.05	-15.85 ± 0.02
31	-9.55	-51.29 ± 0.07	-31.09 ± 0.31	46.50 ± 0.18	-3.89 ± 0.00	-22.49 ± 0.00	-17.28 ± 0.18
33	-9.11	-50.52 ± 0.09	-47.74 ± 0.25	59.83 ± 0.21	-3.80 ± 0.00	-23.09 ± 0.01	-19.13 ± 0.08
34	-9.81	-51.82 ± 0.24	-37.55 ± 0.32	48.71 ± 0.08	-3.67 ± 0.00	-23.16 ± 0.01	-21.17 ± 0.09
36	-9.14	-53.24 ± 0.16	-36.54 ± 0.22	52.61 ± 0.10	-3.91 ± 0.00	-23.41 ± 0.03	-17.67 ± 0.28
37	-9.14	-53.06 ± 0.11	-29.70 ± 0.37	43.72 ± 0.23	-4.12 ± 0.00	-23.47 ± 0.03	-19.69 ± 0.35
38	-8.90	-53.56 ± 0.05	-31.26 ± 0.07	49.38 ± 0.23	-3.86 ± 0.00	-23.03 ± 0.03	-16.27 ± 0.09

References

(1) Case, D. A. et al. Amber 2024. 2024; University of California, San Francisco.

(2) Case, D. A. et al. AmberTools. *J. Chem. Inf. Model.* **2023**, *63*, 6183–6191.

(3) Maier, J. A.; Martinez, C.; Kasavajhala, K.; Wickstrom, L.; Hauser, K. E.; Simmerling, C. ff14SB: Improving the Accuracy of Protein Side Chain and Backbone Parameters from ff99SB. *J. Chem. Theory Comput.* **2015**, *11*, 3696–3713.

(4) Jorgensen, W. L.; Chandrasekhar, J.; Madura, J. D.; Impey, R. W.; Klein, M. L. Comparison of simple potential functions for simulating liquid water. *J. Chem. Phys.* **1983**, *79*, 926–935.

(5) Wang, J.; Wolf, R. M.; Caldwell, J. W.; Kollman, P. A.; Case, D. A. Development and Testing of a General AMBER Force Field. *J. Comput. Chem.* **2004**, *25*, 1157–1174.

(6) He, X.; Man, V. H.; Yang, W.; Lee, T.-S.; Wang, J. A Fast and High-Quality Charge Model for the Next Generation General AMBER Force Field. *J. Chem. Phys.* **2020**, *153*, 114502.

(7) Ryckaert, J.-P.; Ciccotti, G.; Berendsen, H. J. Numerical Integration of the Cartesian Equations of Motion of a System with Constraints: Molecular Dynamics of n-Alkanes. *J. Comput. Phys.* **1977**, *23*, 327–341.

(8) Wang, J. M.; Hou, T. J.; Xu, X. J. Recent Advances in Free Energy Calculations with a Combination of Molecular Mechanics and Continuum Models. *Curr. Comput.-Aided Drug Des.* **2006**, *2*, 287–306.

(9) Wang, J.; Wang, W.; Huo, S.; Lee, M.; Kollman, P. A. Solvation Model Based on Weighted Solvent Accessible Surface Area. *J. Phys. Chem. B* **2001**, *105*, 5055–5067.

(10) Hao, D.; He, X.; Ji, B.; Zhang, S.; Wang, J. How Well Does the Extended Linear Interaction Energy Method Perform in Accurate Binding Free Energy Calculations? *J. Chem. Inf. Model.* **2020**, *60*, 6624–6633.

(11) Abraham, M. J.; Murtola, T.; Schulz, R.; Páll, S.; Smith, J. C.; Hess, B.; Lindahl, E. GROMACS: High performance molecular simulations through multi-level parallelism from laptops to supercomputers. *SoftwareX* **2015**, *1–2*, 19–25.

(12) Kühne, T. D. et al. CP2K: An electronic structure and molecular dynamics software package - Quickstep: Efficient and accurate electronic structure calculations. *J. Chem. Phys.* **2020**, *152*, 194103.
